# The Development of an Oral Solution Containing Nirmatrelvir and Ritonavir and Assessment of Its Pharmacokinetics and Stability

**DOI:** 10.3390/pharmaceutics16010109

**Published:** 2024-01-14

**Authors:** Lili Wang, Zhuang Ding, Zhengping Wang, Yanna Zhao, Hengqian Wu, Qipeng Wei, Lingfeng Gao, Jun Han

**Affiliations:** 1 School of Chemistry and Chemical Engineering, University of Jinan, Jinan 250022, China; 2 Institute of Biopharmaceutical Research, Liaocheng University, Liaocheng 252000, China

**Keywords:** nirmatrelvir, ritonavir, bioavailability, stability, liquid formulation

## Abstract

Paxlovid^®^, a co-packaged medication comprised of separate tablets containing two active ingredients, nirmatrelvir (NRV) and ritonavir (RTV), exhibits good effectiveness against coronavirus disease 2019 (COVID-19). However, the size of the NRV/RTV tablets makes them difficult for some patients to swallow, especially the elderly and those with dysphagia. Therefore, an oral liquid formulation that can overcome this shortcoming and improve patient compliance is required. In this study, we developed a liquid formulation containing NRV and RTV by adopting strategies that used co-solvents and surfactants to enhance the solubility and inhibit possible recrystallization. The in vitro release results showed that NRV and RTV could be maintained at high concentrations in solution for a certain period in the investigated media. In vivo studies in rats showed that the oral bioavailability of NRV/RTV solution was significantly enhanced. Compared to Paxlovid^®^ tablets, the AUC_(0–t)_ of NRV and RTV increased by 6.1 and 3.8 times, respectively, while the C_max_ increased by 5.5 times for both. Furthermore, the promoting effect of the absorption of RTV on the bioavailability of NRV was confirmed. Experiments with a beagle showed a similar trend. Stability studies were also conducted at 4 °C, 25 °C, and 40 °C for 90 days, indicating that the oral liquid formulation was physically and chemically stable. This study can be used as a valuable resource for developing and applying oral liquid NRV/RTV formulations in a clinical context.

## 1. Introduction

Coronavirus disease 2019 (COVID-19), caused by severe acute respiratory syndrome coronavirus 2 (SARS-CoV-2), has caused worldwide concern and poses a serious threat to human health globally, as well as affecting social and economic conditions worldwide [1]. Elderly patients and those with underlying diseases are more likely to develop severe disease and have higher in-hospital mortality rates [2]. Furthermore, the relaxation of restrictions in the context of social liberalization and pandemics increases the risk of serious infectious complications in these individuals [3]. Nirmatrelvir (NRV)/ritonavir (RTV) (Paxlovid^®^ tablet) was developed and marketed by Pfizer and exhibits high efficacy in treating COVID-19 by reducing the risk of disease progression, hospitalization, and mortality [4,5,6]. Treatment of symptomatic COVID-19 with Paxlovid^®^ resulted in an 89% reduction in the risk of progression to severe COVID-19 compared to that with a placebo [6]. According to the global Evaluation of Protease Inhibition for COVID-19 in High-Risk Patients (EPIC-HR) trial, Paxlovid^®^ treatment provided a 94% risk reduction compared to placebo treatment in patients that were 65 years of age or older, and interim data from the trial showed that in infected adults with a low risk of hospitalization or mortality, a 70% decrease in hospitalization and no deaths were observed in the treatment group compared to that in the placebo group [6,7].

Paxlovid^®^ is a co-packed medicinal product containing two active substances in separate pharmaceutical forms: NRV and RTV [8,9]. NRV, the core antiviral ingredient, acts as a peptidomimetic inhibitor of the SARS-CoV-2 main protease (Mpro) [10,11]. RTV is an HIV-1 protease and cytochrome P450 3A4 enzyme (CYP3A4) inhibitor [12]. The standard dose of Paxlovid^®^ is 300 mg NRV (two tablets) and 100 mg RTV (one tablet) twice a day for 5 days (a total of 30 tablets) [4,8,9]. The dimensions of the NRV tablet and RTV tablet are approximately 17.5 mm × 8.5 mm × 5.7 mm and 17.0 mm × 9.0 mm × 6.0 mm (length × width × thickness), respectively [8]. Owing to the large size of NRV and RTV tablets, administration of Paxlovid^®^ as an oral medication increases the risk of choking in the elderly and patients with dysphagia [5]. Specific information on NRV and RTV tablets is provided in Supplementary Materials.

Liquid formulations have advantages over solid formulations [13,14]: (1) Their convenient administration: oral solutions are convenient for patients with dysphagia to ingest the drug directly through the oral cavity without additional handling or burden. (2) Rapid absorption: The drug does not need to be dissolved and can be quickly dispersed and absorbed via the gastrointestinal tract. (3) Adjustable dosage: Liquid formulations can be adjusted according to the patient’s needs to ensure precise dosing. This is particularly important in the therapeutic process, as the need for and tolerance to the drug may vary from patient to patient. (4) Improved solubility: For biopharmaceutical classification system (BCS) class II (low solubility-high permeability) drugs, successful liquid formulations can improve drug efficacy by enhancing solubility and absorption properties [14,15]. However, to our knowledge, no liquid formulation containing both NRV and RTV is currently available, nor are there any studies on an extemporaneously developed formulation. Therefore, there is a need to develop a stable liquid formulation containing NRV and RTV to improve regimen compliance in patients with dysphagia.

The physicochemical properties of NRV and RTV are presented in Table S1 (Supplementary Materials). NRV and RTV are poorly water-soluble drugs and are classified as BCS class II/IV and II drugs, respectively [9,15]. Typically, the poor aqueous solubility of a drug limits its oral bioavailability. There are currently several strategies applied to conquer the challenge of improving drug solubility, including particle size reduction, pre-drug administration, pH adjustment, salt formation, and the addition of surfactants and co-solvents [16]. The addition of co-solvents is one of the most widely used methods. Co-solvents decrease the polarity of water by reducing its intermolecular hydrogen-bonding network, thereby increasing the aqueous solubility of lipophilic drugs [17]. The most commonly used co-solvents include propylene glycol, polyethylene glycol 400, glycerol, and ethanol [18]. 

Liquid formulations based on co-solvents can form supersaturated solutions with concentrations much higher than equilibrium solubility after oral administration in the gastrointestinal tract [19]. This supersaturated solution is a thermodynamically unstable system that tends to crystallize and precipitate, leading to the loss of solubility advantages [20]. Therefore, successfully supersaturated liquid formulations should not only have high drug concentrations but also be able to maintain the supersaturated state. Surfactants not only improve drug solubility but can also act as precipitation inhibitors, thus maintaining a supersaturated state [21]. Surfactants with amphiphilic structures can reduce surface tension, improve drug wettability, and enhance solubility by forming micelles [22]. According to previous reports, surfactants can also affect the crystalline nucleation induction time in aqueous media, promoting or inhibiting crystallization [23,24]. Therefore, the screening of surfactants is necessary for liquid formulations. Reportedly, co-solvents and surfactants have been frequently combined, as evidenced by the ritonavir oral solution (Norvir^®^), lopinavir/ritonavir oral solution (Kaletra^®^), and tipranavir oral solution (Aptivus^®^) (Table S2 in the Supplementary Materials) [25,26,27].

In this study, preformulation studies and formulation screening were carried out to develop an oral solution for NRV/RTV. The performance of different surfactants in improving solubility and inhibiting recrystallization was investigated. The NRV/RTV oral solution formulation was fabricated by the administration of co-solvents and surfactants. Furthermore, in vitro evaluations, in vivo pharmacokinetic studies, and stability studies were performed and reported. These results provided evidence that the NRV/RTV liquid formulation can be an efficient drug delivery system, enhance oral bioavailability, and be beneficial to patients with dysphagia.

## 2. Materials and Methods

### 2.1. Materials

NRV (98.7% purity) was provided by Yangzhou Aurisco Pharmaceuticals Co., Ltd. (Yangzhou, China). RTV (99.8% purity) was supplied by Anhui Biochem United Pharmaceutical Co., Ltd. (Hefei, China). Paxlovid^®^ (NRV/RTV tablet) was acquired from Pfizer Inc. (Chicago, IL, USA). Polysorbate 80 (Tween 80), Polyoxyl (35) hydrogenated castor oil (HCO, Kolliphor^®^ ELP), and Poloxamer 407 (Kolliphor^®^ P 407) were obtained from BASF (Shanghai, China). The telmisartan reference standard (98%) was purchased from Shanghai Aladdin Biochemical Technology Co., Ltd. (Shanghai, China). Ethanol (99.5%) was obtained from Comel Chemical Reagent Company (Tianjin, China). Propylene glycol was obtained from Hunan Erkang Pharmaceutical Co., Ltd. (Changsha, China) and used as the co-solvent. High-performance liquid chromatography (HPLC)-grade acetonitrile, methanol, ammonium acetate, and formic acid were purchased from Thermo Fisher Scientific, Inc. (Waltham, MA, USA). Pure water was obtained from Wahaha Pure Water, Inc., Hangzhou, China. All other chemicals used in this study were analytical reagent grade.

### 2.2. Solubility Studies

The solubility of active pharmaceutical ingredients (API) in water and buffers with various pH values (pH 1.2–pH 7.4) was investigated to guide formulation screening. The equilibrium solubility method was performed based on the saturated shake-flask solubility technique [28]. An excess of each API was added into volumetric flasks containing the medium and placed in a thermostatic shaking chamber (HNY-2102C, Tianjin, China) for 24 h of continuous shaking (150 r/min) at 37 °C. The supernatant was filtered through a 0.45 μm PTFE filter membrane, and then an equal amount of methanol was added to prevent API precipitation. The concentrations were quantified using HPLC. Content analysis was performed using an HPLC system (Thermo Fisher Scientific, Waltham, MA, USA) using methanol:water (88:12, *v*/*v*) as the mobile phase. The injection volume of the filtered sample was 10 µL. Separation was performed using an Agilent C18 column (4.60 mm × 250 mm, 5 μm) with a mobile-phase flow rate of 1.0 mL/min. The detection wavelength was set at 203 nm.

The solubility of NRV in organic solvents, including ethanol, propylene glycol, and ethyl acetate, was tested. In addition, the solubility of RTV in organic solvents was retrieved from the literature [8].

### 2.3. Formulation Screening 

#### 2.3.1. Solubilization Effect of Different Surfactants

The solubility of NRV in solutions containing different surfactants was investigated to evaluate the solubilization effects of various surfactants on NRV and RTV. Briefly, 50 mL of aqueous solutions containing different surfactants (0.2%, *m*/*v*) were prepared, and excess amounts of NRV and RTV powders were added to the above solutions. The suspension was placed in a constant-temperature shaker (HNY-2102C, Tianjin, China) for 24 h with continuous shaking (150 r/min) at 37 °C [28]. The suspension was filtered through a 0.45 μm PTFE membrane, and then an equal amount of methanol solution was added to the filtrate to prevent the precipitation of NRV and RTV. The concentrations were quantified using HPLC according to the method in Section 2.2.

#### 2.3.2. Recrystallization Inhibition Abilities of Different Surfactants

The recrystallization inhibition effects of different surfactants on NRV and RTV were evaluated. The solvent shift method was used to create supersaturation [29]. Briefly, 20 μL of ethanol solution with an initial concentration of NRV 60 mg/mL and RTV 20 mg/mL at room temperature was added to 50 mL of aqueous solution and dissolved with different surfactants. The solution was mixed at 50 r/min and 37 °C. A 3 mL sample of the solution was obtained at predetermined time intervals for measurement and supplemented with an equal volume of fresh medium. Experiments were carried out for 180 min, and NRV and RTV concentrations were quantified using HPLC according to the method described above (Section 2.2). All tests were performed in triplicate.

Particle size measurements were performed at different time points using a Malvern Nano ZSP instrument (Malvern, UK). All tests were performed at 25 °C.

### 2.4. Formulations Preparation

Surfactants with optimal performance were screened by comparing the solubilization and recrystallization inhibition effects of different surfactants on NRV and RTV. Moreover, the proportion of excipients in the marketed oral solution (Norvir^®^) was referenced [27]. The prescribing information was determined and is shown in Table 1. Firstly, the blank solvent was prepared by mixing ethanol, propylene glycol, and polyoxyl (35) hydrogenated castor oil (HCO) with purified water. Then, the quantitative API (NRV 300 mg and RTV 100 mg) was weighed into a glass vial. Next, 7.0 mL of blank solvent was added, mixed well, and sonicated until the API was completely dissolved. The solution was filtered through a 0.22 µm PTFE membrane to obtain a colorless to light yellow and clear solution with concentrations of NRV 42.86 mg/mL and RTV 14.29 mg/mL.

### 2.5. Release under Non-Sink Conditions

The evaluation of formulation stability under non-sink conditions has become an important strategy for assessing the true performance of supersaturated formulations [30,31]. Formulation 1 (F1) and RS (Paxlovid^®^ tablet) (equivalent to 300 mg NRV and 100 mg RTV) were used for evaluation in non-sink-conditioned media. In the experiments, 250 mL of different dissolution media was used, the temperature was set at 37 °C, and the rotational speed was set at 400 r/min. Samples (2 mL) were collected at predetermined time intervals and replenished with equal volumes of fresh medium. The experiments were conducted for 360 min. NRV and RTV were analyzed using HPLC according to the method described in Section 2.2.

### 2.6. In Vivo Pharmacokinetics in Rats

Pharmacokinetic studies were performed in rats to compare the oral bioavailability of F1 and RS. In addition, as shown in Table 2, several liquid formulations containing different levels of NRV and RTV were prepared to investigate the effect of RTV on NRV absorption. Male Sprague-Dawley rats (6–8 weeks, 200 ± 20 g) were purchased from Jinan Pengyue Laboratory Animal Breeding Co., Ltd. (Jinan, China). Rats were acclimated at 25 ± 0.5 °C and 60 ± 5% relative humidity for 7 days while being provided with a standard diet. Then, 25 rats were randomly divided equally into five groups of five rats each. They were fasted overnight before administration, allowed to drink water freely, and fasted for 4 h after administration. These formulations were orally administered intragastrically at a dose of 0.7 mL/kg. After administration, 500 μL blood samples were obtained from the orbital plexus of the animals, and heparin was added to avoid clotting. Blood samples were then immediately centrifuged at 5000 r/min for 5 min to separate the plasma. The plasma was stored at –80 °C until further use.

### 2.7. In Vivo Pharmacokinetics in a Beagle

A healthy adult male beagle weighing approximately 10 kg was purchased from Changzhou Beile Experimental Animal Breeding Co., Ltd. (Changzhou, China), fed commercial dry feed twice daily for 7 days before the experiment, and allowed to drink water ad libitum. The experiment was conducted at Shandong Xinbo Drug Research Co., Ltd. (Dezhou, China). Different formulations were orally administered to the dog separately to investigate their pharmacokinetic characteristics. In stage 1, tablets (1 NRV + ½ RTV) were placed at the root of the dog’s tongue (epiglottis), and approximately 50 mL of water was administered immediately. After a 1-week wash-out period, stage 2 was performed. In stage 2, approximately 3.5 mL of the liquid formulation (F1) was administered, followed immediately by approximately 50 mL of water. One milliliter of blood was obtained from the forelimb vein of the dog at predetermined time points after oral administration. The blood was collected into heparin anticoagulant tubes, centrifuged at approximately 5000 r/min for 10 min, and the plasma was extracted. The plasma obtained at each period was stored in a refrigerator at –80 °C as soon as possible for subsequent measurements.

### 2.8. Quantification of NRV and RTV in Plasma

Plasma samples were analyzed using LC-MS/MS (Waters TQ-S) with telmisartan as the internal standard (IS) [32,33]. Briefly, 400 μL of IS solution (0.25 µg/mL of IS acetonitrile solution) was added to thawed plasma samples at ambient temperature and mixed vigorously by vortexing. Then, the sample was centrifuged at 4 °C (11,000 r/min for 15 min) to remove proteins and obtain the supernatant. The supernatant was further filtered through a 0.22 μm syringe-driven filter prior to analysis.

An ACQUITY UHPLC ^TM^ I-Class system (Waters, Milford, CT, USA) was employed to separate the analytes on a Waters Symmetry^®^ C18 column (2.1 mm × 100 mm, 3.5 μm). The temperature of the column oven and sample tray were set at 40 °C and 10 °C, respectively. Methanol was used as mobile phase A and 2 mM ammonium acetate aqueous solution containing 0.1% formic acid was used as mobile phase B. A total flow rate of 0.2 mL/min was used, and the gradient program was performed as follows: 0–0.5, 95% B; 1.2–2.0 min, 5% B; 2.1–4.0 min, 95% B. The injection volume was set to 1 μL. Data acquisition and quantitative analyses were performed using a Waters TQ-S system (Waters MS Technologies, Manchester, UK). Multiple reaction monitoring (MRM) was employed to analyze the analytes and IS in positive polarity mode with electrospray ionization (ESI).

### 2.9. Storage Stability

The solutions sealed in amber glass vials were placed at 4 °C, 25 °C, and 40 °C to evaluate their physical and chemical stability. Physical stability was determined visually at selected time points by monitoring any changes in sensory properties such as odor, clarity, color, and appearance of precipitate [34]. Chemical stability studies were performed by monitoring residual drug concentrations by HPLC (Section 2.2). The residual concentrations (%) of NRV and RTV were determined using the following equation: Residual concentrations (%) of NRV/RTV = NRV/RTV content on day (n)/NRV/RTV content on day (0) × 100%. The formulation was considered stable if the physical properties did not change and the drug concentration remained between 90% and 110% of the original concentration, as per the guidelines for stability testing [34,35].

The pH was determined using a calibrated pH meter (Mettler Toledo, Five Easy Plus, Greifensee, Switzerland). A Density and Sound Velocity Meter (Anton Paar DSA 5000M, Graz, Austria) was used to determine the density (20 °C). Microbiological tests of the formulations were performed after 90 days using the pour-plate method for seeding [36,37].

### 2.10. Statistical Analysis

Data are expressed as means and standard deviations (SD). Additionally, data were analyzed and plotted using Origin Pro 2023 (OriginLab Inc., Northampton, MA, USA). Pharmacokinetic parameters, such as the area under the curve from zero to last blood sampling time (AUC_(0–t)_), half-life period (*t*_1/2_), peak time (*T*_max_), and peak concentration (*C*_max_), were calculated using Win Nonlin 6.1 software (Pharsight, Inc., Mountain View, CA, USA).

## 3. Results and Discussion

### 3.1. Solubility of NRV and RTV

The solubility analysis results for NRV and RTV in different aqueous systems are presented in Table 3. Based on the solubility results of NRV, it is evident that solubility does not significantly depend on pH (pH 1.2–8.0). The solubility of NRV in each buffer fell within a range of 950–1000 µg/mL. RTV is a weakly basic compound with pH-dependent solubility in various buffer systems [16]. The pKa values of RTV are 2.01 and 2.51, owing to the two weakly basic thiazole moieties [38]. When pH was below 2.0, the ionic component of the RTV solution was greater than the neutral molecular component, leading to a significantly increased solubility. Hence, RTV had a high solubility in pH 1.2 medium (approximately 381.9 µg/mL). However, the pH value of most oral solutions is between 2 and 9, which maintains drug stability and improves the compatibility of the drug with the body [39]. Therefore, it is not feasible to significantly enhance the solubility of the two medications by adjusting the pH value of the solution.

As listed in Table 3, the solubilities of NRV and RTV in water were 953.1 ± 7.6 µg/mL and 3.7 ± 0.5 µg/mL, respectively. RTV is almost insoluble in water, indicating that the poor solubility of RTV should be resolved. The solubilities of NRV and RTV in organic solvents commonly used in oral solutions are shown in Table S3 (Supplementary Materials). The solubility of RTV in ethanol was 165 mg/mL, whereas that in propylene glycol was greater than 200 mg/mL. Hence, ethanol and propylene glycol were selected as co-solvents to prepare the highly concentrated liquid formulations.

### 3.2. Screening of Surfactants

#### 3.2.1. Solubilizing Effect of Different Surfactants

Surfactants can be classified as nonionic, cationic, anionic, or amphoteric, of which nonionic surfactants with low toxicity cause minimal irritation and are widely used as solubilizers, wetting agents, or crystal inhibitors in oral solution formulations [18]. The selection of a surfactant for poor water-soluble drugs is crucial for obtaining high drug loading and preventing drug precipitation as a result of dilution in the gastrointestinal tract [40,41]. Here, several nonionic surfactants, including Tween 80, HCO, and Poloxamer, were evaluated at a concentration of 0.2% (*m*/*v*) to ascertain their ability to enhance the solubility of NRV and RTV.

**Figure 1:**
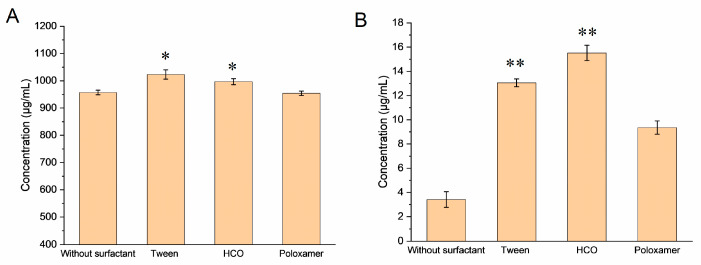
Solubility of nirmatrelvir (NRV) (**A**) and ritonavir (RTV) (**B**) in water with different surfactants (data are presented as the mean ± SD, *n* = 3). * *p <* 0.05 vs. the no surfactant group, and ** *p* < 0.001 vs. the no surfactant group.

According to the results illustrated in Figure 1A, the different surfactants affected NRV solubility to varying degrees, relative to that in the media without polymers. The solubilities of NRV increased in the presence of HCO and Tween (*p* < 0.05), whereas there was no significant change in the solubility of NRV in the presence of poloxamer.

Based on the results presented in Figure 1B, the aqueous solubility equilibrium of RTV in the surfactant-free medium was approximately 3.4 µg/mL. The addition of different surfactants led to varying degrees of improvement in RTV solubility (*p* < 0.001). The increase in the solubility of RTV in surfactants was HCO > Tween > poloxamer. HCO had a superior solubilizing effect on RTV to that of the poloxamer (almost 5-fold higher, *p <* 0.001) and Tween (approximately 1-fold higher).

#### 3.2.2. Inhibition of Recrystallization of NRV/RTV by Different Surfactants

**Figure 2:**
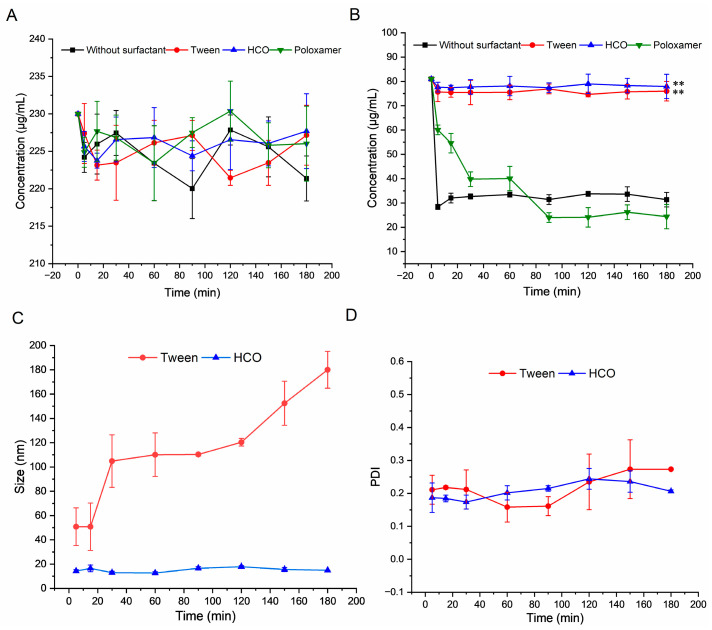
Recrystallization rates of nirmatrelvir (NRV) (**A**), and ritonavir (RTV) (**B**). Particle size (**C**), and particle size distribution (**D**) of solutions of supersaturated NRV/RTV in solutions dissolved with different surfactants (data are presented as the mean ± SD, *n* = 3). ** *p* < 0.001 vs. no surfactant group.

To assess the ability of surfactants to sustain the supersaturated state, changes in the NRV/RTV concentration and particle size in diverse aqueous solutions of pre-dissolved surfactants were observed. The results in Figure 2A show that there was no significant alteration in NRV concentration compared to the original concentration. 

As presented in Figure 2B, surfactants showed significantly different recrystallization inhibition abilities compared to those of the group without surfactants. The concentration of RTV was effectively sustained during the experimental period following the addition of HCO and Tween (*p* < 0.001). However, recrystallization inhibition was more notable in the HCO group, which is consistent with the results of the solubility enhancement experiments. The addition of the surfactants HCO and Tween facilitated micellization of the system, resulting in enhanced recrystallization inhibition in the supersaturated solution of RTV [42]. The impact of surfactants on drug crystallization in saturated solutions is multifaceted, including the crystalline nucleation induction time and desaturation rates [43].

The results of the evaluation of particle size, depicted in Figure 2C,D, demonstrate that the particles in the medium with HCO were smaller and more uniformly dispersed than those in the other surfactant solutions. HCO likely formed micelles of uniform size that encapsulated RTV, resulting in solubility enhancement and recrystallization inhibition. Therefore, HCO was deemed a suitable surfactant for the preparation of NRV/RTV liquid formulation, specifically in the context of supersaturated cosolvent delivery systems. This was consistent with the surfactants used in the marked RTV oral solutions [27]. The particle characteristics in the poloxamer and surfactant-free groups were not obtained because their size (>5 µm) was too large, resulting in poor measurement repeatability.

### 3.3. Release under Non-Sink Conditions

Oral supersaturation systems consist of liquid formulations that are highly concentrated and exposed to gastrointestinal fluids, which leads to the formation of supersaturated solutions owing to the dilution effect of the gastrointestinal fluids [31]. These supersaturated states are thermodynamically unstable and tend to precipitate rapidly in vivo before being absorbed, resulting in compromised bioavailability [44]. Therefore, maintaining a supersaturated concentration of the drug in the body to prevent it from precipitating before absorption is crucial for designing supersaturated formulations [45]. Employing dissolution methods under non-sink conditions has emerged as a crucial approach for determining the actual performance of supersaturated formulations [30,31]. In this study, the formulation was assessed in vitro in various media, including water, acid solution (pH 1.2), acetate buffer (pH 4.5), and phosphate buffer (pH 6.8), to evaluate the maintenance of supersaturated solubility [46]. In addition, as the in vitro evaluation experiment served as a preliminary evaluation, and the focus of this study was the in vivo experiment, multiple repeated experiments were deemed unnecessary.

**Figure 3:**
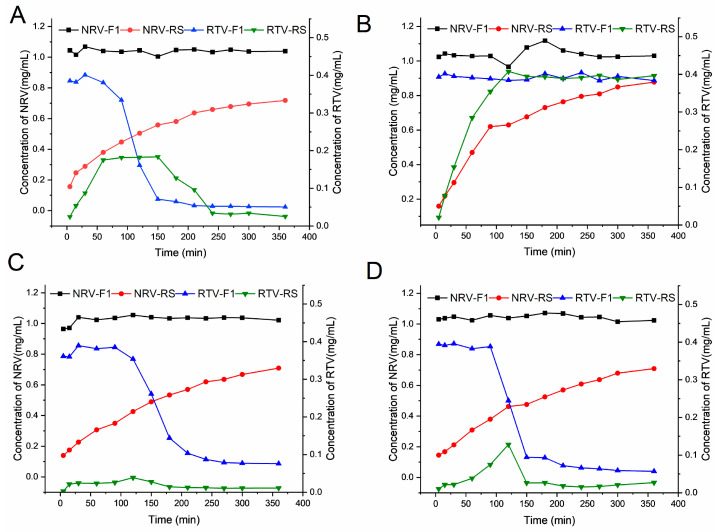
Changes in the concentrations of nirmatrelvir/ritonavir (NRV/RTV) for F1 and RS in media with various pH values: (**A**) water; (**B**) pH 1.2; (**C**) pH 4.5; and (**D**) pH 6.8.

As shown in Figure 3, NRV solubility in the liquid formulation F1 remained high (~1.0 mg/mL) when added to various media (water, pH 1.2, 4.5, and 6.8) with minimal alterations. This can be ascribed to the addition of surfactants and organic solvents increasing the NRV supersaturation concentration in F1 [47]. However, RS, as a tablet form, showed a process of disintegration and dissolution in all media studied. Therefore, NRV exhibited a consistent release pattern, with an incremental increase in release over time for RS. RS showed a lower NRV dissolution rate compared to F1, with the maximum dissolution rate reaching only 60% of F1 in water, pH 4.5, and pH 6.8 media, and only 80% of F1 in pH 1.2 media. According to the Noyes–Whitney equation, the dissolution rate of the drug is directly proportional to the equilibrium solubility of the drug in the solvent and the surface area of dissolution and is inversely proportional to the solvent volume [31,48]. Hence, we speculate that the lower dissolution rate of NRV for RS may be due to the influence of solvent volume or supersaturated solubility. Furthermore, it was observed that, in comparison to other media, the dissolution rate of NRV in pH 1.2 media was higher for RS. This could be attributed to the complete dissolution of RTV at pH 1.2 (Figure 3B). The dissolution behavior of RTV (an amorphous solid dispersion formulation) in other media exhibits two modes: Dissolution and precipitation [31]. During the precipitation process, a certain amount of NRV may be encapsulated, which limits its dissolution [49].

As demonstrated in Figure 3A,C,D, the concentration of RTV for F1 in water, pH 4.5, and pH 6.8, was maintained at supersaturation levels for 90 min. Subsequently, the concentration began to decrease, ultimately stabilizing after 150–200 min (0.01–0.04 mg/mL). As seen in Figure 3B, in pH 1.2 medium, RS was fully released after 100 min owing to the higher solubility of RTV at pH 1.2 (as described in Section 3.1). As shown in Figure 3C,D, RS released approximately 10% and 30% of RTV in pH 4.5 and pH 6.8 media over 120 min, respectively, compared with that of F1. Following this, the concentration gradually declined to a final concentration of 0.01–0.02 mg/mL. The dissolution behavior of RS in water is presented in Figure 3A, with 50% of RTV being released after 60 min, followed by a stabilization period of approximately 100 min. This phenomenon was attributed to the preparation of the RTV tablet, which used hot-melt technology to achieve a solid dispersion [9]. Theoretically, the solubility of amorphous RTV is less than 0.02 mg/mL [50]. Previous studies have demonstrated that liquid–liquid phase separation (LLPS) occurs during the dissolution of amorphous solid dispersions [51]. This occurs when the concentration is equal to the amorphous solubility, and the drug remains in a supersaturated state until crystallization occurs. The concentration subsequently decreases until it reaches the solubility of the crystal [52]. 

The variation in the concentrations of the two forms in all media under non-sink conditions indicated that the liquid formulation (F1) resulted in much higher concentrations of NRV and RTV than those from RS up to 100 min (Figure 3). 

### 3.4. In Vivo Pharmacokinetics in Rats

**Figure 4:**
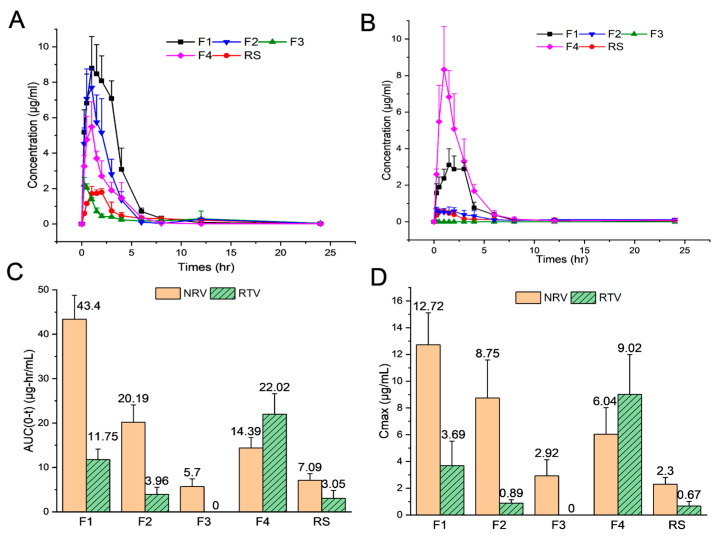
Concentration-time profiles of nirmatrelvir (NRV) (**A**) and ritonavir (RTV) (**B**), and AUC_(0–t)_ (**C**) and *C*_max_ (**D**) of values of NRV/RTV in rat plasma after oral administration of F1 (RS-equivalent specification), F2 (half the amount of RTV relative to that of RS), F3 (no RTV), F4 (half the amount of NRV relative to that of RS), and RS. Data are presented as the mean ± SD, *n* = 5.

F1 (RS-equivalent specification), F2 (half the amount of RTV relative to that of RS), F3 (no RTV), F4 (half the amount of NRV relative to that of RS), and Paxlovid^®^ (RS) were further studied to determine their pharmacokinetic behavior in vivo. This was performed to compare the pharmacokinetic parameters of the liquid formulation with those of RS in rats and to investigate the influence of RTV on the absorption of NRV. According to the data presented in Figure 4 and Table 4, there was a substantial difference in the *C*_max_ and AUC_(0–t)_ between the five formulations.

As shown in Figure 4C,D, and Table 4, F1 exhibited considerably higher plasma concentrations of NRV/RTV at an equal dose. The AUC_(0–t)_ of NRV and RTV increased by 6.1- and 3.8-fold, respectively, while C_max_ increased 5.5-fold for both, compared to those for RS. Additionally, the *T*_max_ of NRV/RTV in F1 was lower than that of RS, whereas there was no significant difference in *t*_1/2_ between the two formulations (Table 4). These findings indicated that F1 improved the absorption rate of the drug but did not substantially affect its metabolism. Furthermore, the liquid formulation F1 led to greater plasma concentrations of NRV/RTV in the short term than RS. This can be attributed to the rapid dispersion and absorption of the liquid formulation. Besides, this observation was consistent with the results of the in vitro experiments.

In comparison with those of RS, the *C*_max_ and AUC_(0–t)_ of NRV for F2 (half the amount of RTV relative to that of RS) increased by 3.8- and 2.8-fold, respectively, whereas those of F4 (half the amount of NRV relative to that of RS) increased by 2.6-fold and 2.0-fold, respectively (Figure 4C,D, Table 4). This suggests that, compared to RS, the liquid formulations with a lower dosage of NRV and/or RTV achieved enhanced bioavailability. These results showed that a lower-dose liquid formulation can be developed to reduce the possibility of drug side effects, but this needs to be verified in human clinical trials.

Furthermore, F1, F2, and F3 were studied to acquire precise information on how RTV promotes NRV absorption (Figure 4A–D, Table 4). The only variation among these formulations was the amount of RTV administered. Remarkable differences were observed in the *C*_max_ and AUC_(0–t)_ of NRV between all three formulations. Compared to F1, F2 (with half the amount of RTV) showed a 1.4-fold decrease in *C*_max_ and a 2.1-fold decrease in AUC_(0–t)_. However, the C_max_ and AUC_(0–t)_ of NRV in RTV-free F3 were 4.3-fold and 7.6-fold lower, respectively, in comparison with F1. The ranking of the *C*_max_ and AUC_(0–t)_ of NRV among the three prescriptions was F1 > F2 > F3, indicating the significant contribution of RTV to the absorption and bioavailability of NRV. The potentiating effect of RTV is due to the effective inhibition of cytochrome P450 3A (CYP3A) [53]. This reduces the enzymatic breakdown of NRV, leading to altered pharmacokinetic parameters, such as AUC_(0–t)_ and *C*_max_ [53]. Although RTV is inactive against SARS-CoV-2 Mpro, it can maintain NRV at a higher concentration in the body for an extended period [12]. In addition, we notice that the AUC_(0–t)_ of RTV for F4 is approximately twice that for F1. There may be various reasons for this. One possibility is the competition between two poorly soluble drugs for co-solvents and solubilizers. Another possibility is that the absorption of NRV may affect that of RTV. Currently, the primary cause is unclear.

### 3.5. In Vivo Pharmacokinetics in a Beagle

**Figure 5:**
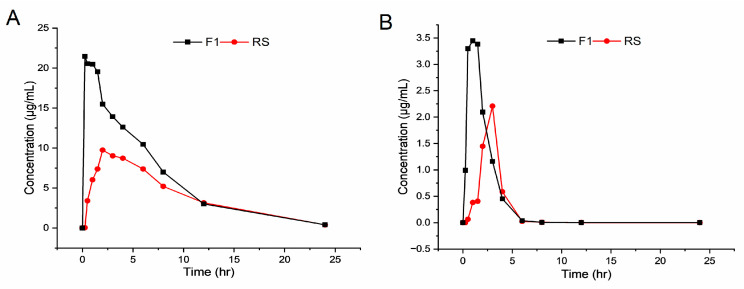
Concentration–time profiles of nirmatrelvir (NRV) (**A**) and ritonavir (RTV) (**B**) in the plasma of a beagle after oral administration of F1 and RS (Paxlovid^®^) (*n* = 1).

An in vivo study was performed in a beagle to further validate the bioavailability of the liquid formulation. After a single oral administration of 150 mg NRV/50 mg RTV, the plasma concentrations of NRV and RTV were determined using a validated LC-MS/MS method. Figure 5 shows the mean plasma concentrations of NRV and RTV for F1 and RS. The results indicated that F1 was absorbed at a faster rate than RS, as evidenced by the plasma concentrations of NRV and RTV. This phenomenon could potentially be attributed to the swift dispersal of the drugs in the liquid formulation (F1) and the dissolution behavior of the tablet (RS). The primary pharmacokinetic parameters, including AUC_(0–t)_, *C*_max_, *T*_max_, and *t*_1/2_, for both products, are presented in Table 5. The AUC_(0–t)_ (174.55 µg·h/mL) and *C*_max_ (25.76 µg/mL) of NRV for the test liquid F1 were approximately two times higher than those of RS (95.36 µg·h/mL and 9.75 µg/mL). Plasma concentrations of NRV reached *C*_max_ faster for F1 (*T*_max_ = 0.25 h) than for RS (*T*_max_ =2 h).

Based on the pharmacokinetic parameters of RTV (Figure 5B and Table 5), F1 had significantly higher *C*_max_ and AUC_(0–t)_ values (3.45 µg/mL and 8.44 µg·h/mL) compared to those of RS (2.21 µg/mL and 4.68 µg·h/mL). In addition, the *t*_1/2_ parameter of RTV for F1 was 3.77 h, and for RS was 2.05 h, suggesting that the metabolism of RTV may be faster in liquid form. However, this may require multiple, repeated experiments to verify. 

In conclusion, liquid formulations significantly improved the oral bioavailability of NRV and RTV, suggesting that similar improvements may be expected in human trials.

### 3.6. Stability Studies

**Figure 6:**
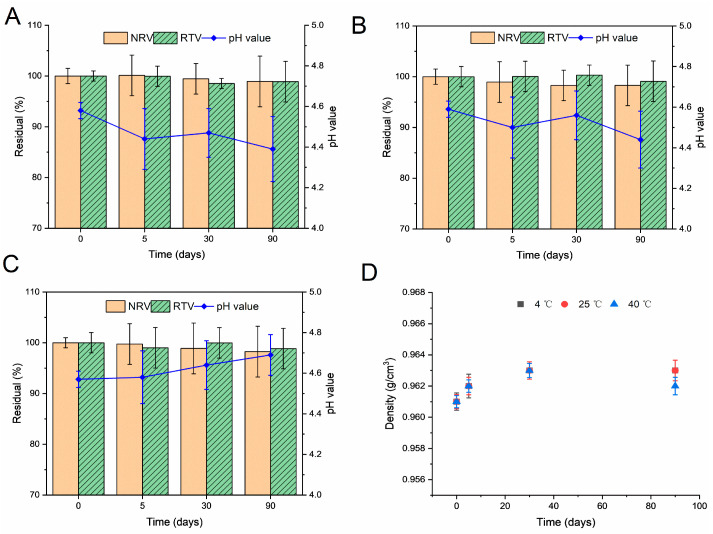
Stability of the liquid formulation after storage at 4 °C (**A**), 25 °C (**B**), and 40 °C (**C**), determined by the residual concentrations (%) of nirmatrelvir (NRV) and ritonavir (RTV), and density (**D**).

Chemical and physical stability studies were conducted on samples that were stored in glass vials and protected from light at temperatures of 4 °C, 25 °C, and 40 °C for a duration of 3 months. The samples remained clear throughout the study, with no changes in color, clarity, or odor. No suspended particles or precipitates were observed. Furthermore, as indicated in Figure 6A–C, there were no significant changes in the content of NRV and RTV over time during storage. As stated above, the physical and chemical stabilities of the solution remained unaffected under the investigated storage conditions.

Secretan et al. reported that NRV was stable under oxidative and photolytic conditions but hydrolytic under strongly acidic and alkaline conditions because of the amide moieties and nitrile function group [54]. Therefore, in this study, it was necessary to control the pH of the solution. The pH measurements shown in Figure 6A–C indicate that the formulations demonstrated minimal pH changes throughout the study period, with values ranging from 4.3 to 4.8. No significant changes were observed between the three storage environments. In addition, the total colony counts met the pharmacopeial specifications, and no *E. coli* growth was detected after 90 days [36,37]. 

Figure 6D shows that there was no considerable alteration in the formulation density at each temperature (change rate of approximately < 0.2%). The current results collectively indicate that the oral liquid formulation maintains physical, chemical, and microbial stability for 90 days at temperatures of 4 °C, 25 °C, and 40 °C, which is satisfactory. However, further investigation of impurity analysis in the formulation is needed in the future.

## 4. Conclusions

In this study, an oral solution of NRV/RTV with adequate physical and chemical stability was prepared by combining co-solvents and surfactants to enhance the equilibrium solubility of NRV/RTV and prevent recrystallization in aqueous media. HCO was used as an effective solubilizer and crystallization inhibitor. Evaluations conducted under non-sink conditions demonstrated that liquid form F1 produced considerably higher concentrations of NRV and RTV compared to RS for up to 100 min.

Furthermore, the results from pharmacokinetic studies in rats and a dog showed that the bioavailability of NRV was significantly enhanced in the liquid formulation compared to that of the RS, Paxlovid^®^. Additionally, this study provides precise information on the effect of the concentration of RTV on NRV absorption and subsequent bioavailability. The limitation of this study is the lack of data from human trials. However, these findings are a valuable resource for developing and applying oral liquid NRV formulations in a clinical context.

## Figures and Tables

**Table 1:** Composition of formulation 1 (7.0 mL).

Components	Quantities	Functional Category
NRV	300 mg	API
RTV	100 mg	API
Ethanol	3.0 mL	Co-solvent
Propylene glycol	1.9 mL	Co-solvent
Surfactant	0.7 mL	Solubilizer
Water	1.40 mL	Solvent

API: active pharmaceutical ingredient.

**Table 2:** Composition of each tested formulation for in-vivo studies in rats.

Components	F1 ^a^	F2 ^a^	F3 ^a^	F4 ^a^	RS ^b^
Dose of NRV (mg)	300	300	300	150	300
Dose of RTV (mg)	100	50	0	100	100
Blank solvent (mL)	7.0	7.0	7.0	7.0	–

F1: Formulation 1 (RS-equivalent specification); F2: formulation 2 (half the amount of RTV relative to that of RS); F3: formulation 3 (no RTV); F4: formulation 4 (half the amount of NRV relative to that of RS); RS: Paxlovid^®^ tablet; –: no data. ^a^ F1–F4 were administered in the homemade liquid formulation administration groups, and the preparation method is described in Section 2.4. ^b^ RS was prepared as follows: NRV and RTV tablets were ground into bulk powder, and the corresponding doses of the two tablet powders were mixed well and dispersed evenly in 7.0 mL water.

**Table 3:** Solubility of NRV and RTV in aqueous solutions with various pH values.

	Concentration of NRV (Mean ± SD, µg/mL)	Concentration of RTV (Mean ± SD, µg/mL)
pH 1.2	997.7 ± 6.5	381.9 ± 5.8
pH 2.0	983.2 ± 5.4	17.5 ± 0.7
pH 3.8	974.7 ± 4.8	3.6 ± 0.3
pH 4.5	973.5 ± 5.3	3.6 ± 0.4
pH 6.8	957.8 ± 7.5	3.3 ± 0.5
pH 7.4	998.8 ± 6.2	3.4 ± 0.6

**Table 4:** Pharmacokinetic parameters of NRV/RTV in rats after a single dose oral administration of different formulations.

Parameters	NRV					RTV				
	F1	F2	F3	F4	RS	F1	F2	F3	F4	RS
*C*_max_ (µg/mL)	12.72 ± 2.41	8.75 ± 3.83	2.92 ± 1.22	6.04 ± 1.98	2.30 ± 0.51	3.69 ± 1.83	0.89 ± 0.25	0	9.02 ± 2.97	0.67 ± 0.35
*T*_max_ (h)	1.50 ± 0.39	0.95 ± 0.67	0.63 ± 0.43	0.75 ± 0.35	2.38 ± 0.43	2.00 ± 0.97	1.50 ± 0.50	0	1.60 ± 1.39	2.30 ± 1.21
*t*_1/2_ (h)	4.21 ± 1.14	3.57 ± 0.75	2.38 ± 1.19	2.42 ± 0.38	4.52 ± 1.21	9.26 ± 1.83	11.95 ± 4.35	0	4.92 ± 3.09	8.84 ± 2.17
AUC_(0–t)_ (µg·h/mL)	43.40 ± 5.39	20.19 ± 3.91	5.70 ± 1.74	14.39 ± 2.37	7.09 ± 1.51	11.75 ± 2.38	3.96 ± 1.59	0	22.02 ± 4.59	3.05 ± 1.78

F1: Formulation 1 (RS-equivalent specification); F2: formulation 2 (half the amount of RTV relative to that of RS); F3: formulation 3 (no RTV); F4: formulation 4 (half the amount of NRV relative to that of RS); RS: Paxlovid^®^ tablet; AUC_(0–t)_: area under the curve (from zero to last blood sampling time); *t*_1/2_: half-life period; *T*_max_: peak time; *C*_max_: peak concentration. Data are presented as the mean ± SD, *n* = 5.

**Table 5:** Pharmacokinetic parameters of NRV/RTV in a beagle after a single dose oral administration of F1 and RS.

Parameters	NRV		RTV	
	F1	RS	F1	RS
*C*_max_ (µg/mL)	25.76	9.75	3.45	2.21
*T*_max_ (h)	0.25	2.00	1.00	3.00
*t*_1/2_ (h)	3.64	4.27	2.05	3.77
AUC_(0–t)_ (µg·h/mL)	174.55	95.36	8.44	4.68

## Data Availability

The data presented in this study are available in this article (and Supplementary Materials).

## Supplementary Materials

The following supporting information can be downloaded at: https://www.mdpi.com/article/10.3390/pharmaceutics16010109/s1, Table S1: The basic physical and chemical properties of NRV and RTV; Table S2: Selected marketed products containing cosolvent-based systems; Table S3: Solubility of NRV and RTV in different organic solvents.
