# Supplementary material for: The Development of an Oral Solution Containing Nirmatrelvir and Ritonavir and Assessment of Its Pharmacokinetics and Stability
Source: Pharmaceutics. 2024 Jan 14;16(1):109. doi: 10.3390/pharmaceutics16010109 (PMC10818454; doi:10.3390/pharmaceutics16010109)
Supplement: Supplementary file 1 [file pharmaceutics-16-00109-s001.zip › pharmaceutics-2764317-supplementary.pdf]

Supplementary Material:

The development of an oral solution containing nirmatrelvir and ritonavir and assessment of its pharmacokinetics and stability

Lili Wang, Zhuang Ding, Zhengping Wang, Yanna Zhao, Hengqian Wu, Qipeng Wei, Lingfeng Gao, Jun Han

Table S1. The basic physical and chemical properties of NRV and RTV [1–3].

Generic Name	NRV	RTV
Chemical name	(1R,2S,5S)-N-((1S)-1-Cyano-2-((3S)-2-oxopyrrolidin-3-yl) ethyl)-3-((2S)-3,3-dimethyl-2-(2,2,2-trifluoroacetamido) butanoyl)-6,6-dimethyl-3-azabicyclo [3.1.0] hexane-2-carboxamide	10-Hydroxy-2-methyl-5-(1-methylethyl)-1-[2-(1methylethyl)-4-thiazolyl]-3,6-dioxo-8,11-bis(phenylmethyl)-2,4,7,12-tetraazatridecan-13-oic acid, 5-thiazolylmethyl ester, [5S-(5R*,8R*,10R*,11R*)]
CAS	2628280-40-8	155213-67-5
Chemical Formula	$C_{23}H_{32}F_3N_5O_4$	$C_{37}H_{48}N_6O_5S_2$
Molecular Weight	499.54	720.95
State	A non-hygroscopic, white to pale-colored crystalline powder	A non-hygroscopic, a white to light tan powder
Structure	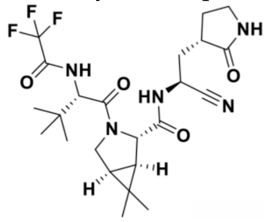	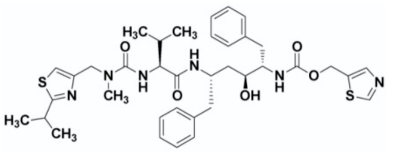
Solubility	It has low solubility in water and buffered aqueous media.	It is almost insoluble in water.
pKa	–	2.01 and 2.51
Melting point	about 192 °C	about 123 °C
Logp	0.845(predictive)	The partition coefficient in the n-octanol-phosphate buffer (0.05 mol/L, pH = 7.4) system was 4.7×10^4
BCS classification	BCS II / IV	BCS II

		Shape			Description	Manufacture
		front	back	side		
Paxovid®	NRV tablet	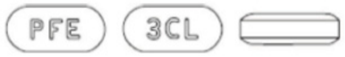			A pink film-coated tablet	Dry granulation
		length 17.5 mm	width 8.5 mm	thickness 5.7 mm		
	RTV tablet	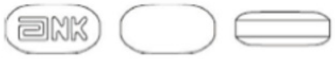			A white to off-white, film-coated tablet	Hot melt extrusion，amorphous solid dispersion
		length 17.1 mm	width 9.1 mm	thickness 6 mm		
		**Figure** 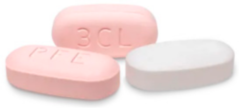				

BCS: biopharmaceutical classification system; NRV: nirmatrelvir; RTV: ritonavir; –: no data.

Table S2. Selected marketed products containing cosolvent-based systems [4].

Drug substance	BCS	Trade name	Indication	Company	Formulation
Ritonavir	Π	Norvir®	HIV infections	AbbVie	ethanol, water, polyoxyl 35 castor oil, propylene glycol
Lopinavir / Ritonavir	Π / Π	Kaletra®	HIV infections	AbbVie	ethanol, propylene glycol, polyoxyl 40 hydrogenated castor oil, povidone
Tipranavir	Π	Aptivus®	HIV antiviral	Boehringer Ingelheim	polyethylene glycol, vitamin E polyethylene glycol succinate, water, propylene glycol
Cyclosporine	Π	Neoral®	Anti-immune	Novartis	alcohol, propylene glycol, corn oil-mono-di-triglycerides, polyoxyl 40 hydrogenated castor oil, DL-α -tocopherol
Amprenavir	Π	Agenerase®	HIV antiviral	GSK	polyethylene glycol 400, propylene glycol, vitamin E polyethylene glycol succinate
Nimodipine	Π	Nymalize®	Hypotensive	Azurity	ethanol, glycerin, methylparaben, polyethylene glycol 400

BCS: biopharmaceutical classification system.

Table S3. Solubility of NRV and RTV in different organic solvents.

	**Concentration of NRV (mg/mL) ***	**Concentration of RTV (mg/mL) [1].**
Ethanol	16.5＜S＜33	165
Ethyl acetate	＞57	1.5
Propylene glycol	＜1.6	＞200

* Crude concentration.

References

1. J.P. Pharmaceuticals and Medical Devices Agency, The interview form of Paxlovid®. Available online: https://www.pmda.go.jp/PmdaSearch/iyakuDetail/GeneralList/62501B5 (accessed on 07 October 2023).
2. EMA European Medicines Agency, Overview of Paxlovid®. Available online: https://www.ema.europa.eu/en/medicines/human/EPAR/paxlovid (accessed on 05 October 2023).
3. Wu, H.; Wang, Z.; Zhao, Y.; Gao, Y.; Wang, L.; Zhang, H.; Bu, R.; Ding, Z.; Han, J. Effect of Different Seed Crystals on the Supersaturation State of Ritonavir Tablets Prepared by Hot-Melt Extrusion. *Eur. J. Pharm. Sci.* **2023**, *185*, 106440.
4. U.S. Food and Drug Administration, Paxlovid® (nirmatrelvir co-packaged with ritonavir) for the treatment of mild-to-moderate coronavirus disease 2019 (COVID-19) in certain adults and pediatric patients. Available online: https://www.fda.gov/media/155049/download (accessed on 08 October 2023).
